# A Tensile Constitutive Relationship and a Finite Element Model of Electrospun Nanofibrous Mats

**DOI:** 10.3390/nano8010029

**Published:** 2018-01-08

**Authors:** Yunlei Yin, Zhongxiang Pan, Jie Xiong

**Affiliations:** 1 School of Materials and Textiles, Zhejiang Sci-Tech University, Hangzhou 310018, China; panzx@zstu.edu.cn; 2 Key Laboratory of Advanced Textile Materials and Manufacturing Technology of Ministry of Education, Zhejiang Sci-Tech University, Hangzhou 310018, China

**Keywords:** electrospun, microstructures, mechanical behavior, finite element model

## Abstract

It is difficult to establish a numerical model for a certain structure of electrospun nanofibrous mats, due to their high porosity and non-linear characteristics, that can fully consider these characteristics and describe their mechanical behaviors. In this paper, an analytical method of meso-mechanics was adopted to establish the tensile constitutive relationship between a single fiber and mats from fiber-web microstructures. Meanwhile, a macroscopic finite element model was developed and verified through uniaxial tensile stress-strain experimental data of silk fibroin (SF)/polycaprolactone (PCL) nanofibrous mats. The compared results show that the constitutive relation and finite element model could satisfactorily express elastic-plastic tensile mechanical behaviors of the polymer. This model helps regulate the microstructure of nanofibrous mats to meet the mechanical requirements in engineering applications.

## 1. Introduction

Electrospinning involves the use of a high-voltage power supply to eject a polymer fiber from a solution and deposit it onto a grounded target. Electrospinning facilitates the optimization of the size, shape, and orientation of fibers through the variation of spinning parameters. Adjusting these variables produces electrospun nanofibrous mats that are suitable for use in the biomedical fields (including artificial organs [1,2], tissue engineering [3,4], and drug delivery systems [5,6]). Electrospun fibrous mats create complex deformation and damaging behavior that is linked to the randomness of their microstructure and the properties of the constituent fibers. Thus, a clear understanding of the mechanical properties that result from individual fiber properties and the microstructure of the networks at both microscopic and macroscopic levels is vital in the design of these materials.

In the last 20 years, many efforts have been made by researchers to understand the performance of electrospun nanofibrous mats. One of the challenges preventing this understanding is the link of the properties of nanofibers and mats’ microstructure to the main mechanisms of the deformation and failure of materials. Experimental characterization is not always viable and sufficient for a comprehensive understanding of the complex phenomena involved in the deformation and damage of electrospun nanofibrous mats. The challenges involved in experimentation are related to the need for specialized experimental devices as well as to the significant efforts required for experimentation. This is especially the case for this type of material, for which mechanical properties are defined by their non-trivial microstructure and the properties of the constituent fibers. To tailor and optimize properties of these materials, an understanding of the relationship between their macroscopic behavior and microstructure and a definition of the manufacturing properties of single fibers is essential. Therefore, the aim of this work is to develop a numerical model incorporating the properties of fibers, the microstructure of mats, and their deformation and damage mechanisms. Such an approach would dramatically reduce experimental effort and would potentially open the door to automatized optimization schemes [7]. However, this critically depends on the availability of a computationally efficient mechanical model, which accurately predicts the mats’ behavior on the different length scales of interest.

Single nanofiber testing is not an easy task due to sample preparation process and the highly sophisticated test devices. High accuracy is required, and external disturbances will skew test results [8,9,10]. Nevertheless, researchers have developed certain methods to test the mechanical properties of a single micro/nanofiber [11,12,13,14]. Generally, these methods were developed to measure the mechanical properties of nanotubes and metal wires. However, they are also suitable for electrospun nanofibrous materials. Among all these tests, the tensile test is the most direct way to measure mechanical properties.

Modeling electrospun nanofibrous mats is also difficult because of the randomness of the meso-structure of the fiber mats and its sophisticated mechanical response. These responses often involve large deformations, rotations, bonding, fiber fracture, fiber slippage, and the continuous recombination of fiber network topology [15]. Cox [16] developed the first non-woven material analysis model. This pioneering research dealt with the mechanical behavior of cellulosic paper. The model calculated the elastic constants of an elastic, long-fiber random network using a small deformation framework. Cox assumed that the fibers were continuous filaments, with their elongation running through the entire structure, while the fiber deformation included only axial deformation. Bending deformation and other fiber behaviors were not fully integrated into the model. Researchers have since proposed several other fibrous mat models, but these models have their limitations [17,18,19,20,21,22]. In view of the complexity of the microstructure of fibrous mats, a representative volume element (RVE) was used to analyze the mechanical behavior of fibrous mats [23,24,25]. Petterson [26] was the first to apply this method to the study of non-woven materials, where the fibers were considered to be a continuum that was straight and elastic. The orientation of fibers conformed to a statistical distribution mode and the bonding points of non-woven materials were rigid. The force required to deform the fabric was absorbed by the fibers in each unit cell. After this, an affine transformation occurred, which contributed to the fiber stress that gathered at the fabric corners and formed stress that is orthogonal to any plane. The fibrous mat model analyzed the post-yield properties of the fibers and predicted the weakest link in the fiber cell, which, with the characteristics, distribution, and orientation of the fibers, determined the elasticity and post-yield performance of non-woven materials. Silberstein et al. [27] suggested employing a similar RVE-based technique to predict the macroscopic behavior of the fabric. Such a model consists of a multi-layer triangular network and uses a homogenization technique to predict a response to monotonic and cyclic loading. These models did not predict the localization of damage or changes in the material’s microstructures. In order to overcome these shortcomings, microstructure-based models employing the direct introduction of individual fibers according to their orientation distribution were developed [28,29,30,31,32,33]. Although this modeling technique is computationally not as efficient as the previous continuum one, it can account explicitly for all the main mechanisms involved in deformation and fracture of non-woven materials. A model that only considers fiber orientation is not sufficient for the prediction of the tensile strength of an individual fiber in random fibrous mats. First of all, the porosity of a mat can significantly alter test results, even if the mat is composed of identical nanofibers. Second, test results can vary when the sample dimensions of a nanofibrous mat specimen vary due to a change in the number of fibers in the specimen, which is involved in resisting deformation under tension. Finally, the morphology of fibers in an electrospun nanofibrous mat has a certain curvature. These above methods cannot predict deformation and damage in terms of the progressive failure of fibers while the realistic material’s microstructure is explicitly incorporated.

In this study, amorphous silk fibroin (SF)/polycaprolactone (PCL) nanofibrous mats were used as a model system because they have exhibited significant cell adhesion and proliferation when used as tissue engineering scaffolds. They can also provide sufficient mechanical strength and toughness to assist in the cell adhesion, growth, migration, and deposition of an extracellular matrix [34,35]. The morphology of the mats was characterized with thermal field emission scanning electron microscopy (FE-SEM) and multi-layer image fusion technology. Structural and topological information for informing models of electrospun nanofibrous mats can be obtained from both FE-SEM of real fiber mats and statistical theories on fiber networks. The microscopy typically provides topological parameters, such as fiber diameter, orientation, and curvature [36,37,38]. The mechanical properties of mats and constituent single fibers were measured under uniaxial tension.

**Figure 1:**
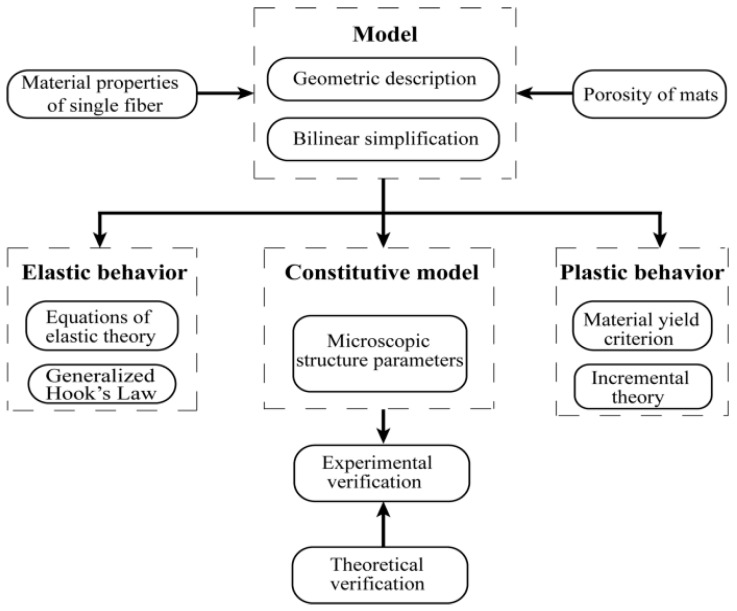
Methodology for the micromechanics constitutive model.

The microstructure of electrospun nanofibrous mats is similar to that of nonwoven fabric. However, the former is special in that there is no adhesion between the connecting points of the fibers, so the interaction force between fibers can be neglected. In this study, electrospun nanofibrous mats with its actual microstructure were modeled here in a finite element environment using a parametric modeling technique. The nature of the electrospun nanofibrous mats was captured by introducing fibers directly into the model according to their orientation distribution in mats. The variability of elastic–plastic mechanical properties of constituent fibers was also introduced. The precision of the model was assessed in comparison to tensile loading experiments under different conditions (the fiber volume fraction, the dimensions of the test specimen, and the fiber curvature). The methodology for the micromechanics constitutive model is shown in Figure 1.

## 2. Materials and Methods

### 2.1. Materials

PCL (8 × 10^4^ g mol^−1^) was purchased from Guanghua Weiye Co., Ltd. (Shenzhen, China). The extraction of regenerated SF has been described by Yin et al. [39]. A 6 wt % solution of the two polymers of SF and PCL (mass ratio 3:2) were dissolved in 1,1,1,3,3,3-hexa flouro-2-propanol (99.5%, Yancheng Dongyang Biological Products Co., Ltd., Yancheng, China). The solution was stirred for 48 h on a magnetic stirrer to produce the spinning solution. The flow rate, plate-to-plate distance, voltage, environment temperature, and relative humidity were set to 1.2 mL·h^−1^, 12 cm, 15 kV, 25 ± 2 °C, and 35 ± 5%, respectively. The nanofibrous mats were collected on an electrically grounded sheet of silver paper. Several single fibers produced under the same conditions were collected on paper templates.

### 2.2. Morphological Characterization

The diameters of nanofibrous materials were determined by analysis of FE-SEM images. Briefly, electrospun mats were mounted onto studs and sputter-coated with a 4–5 nm layer of gold using a Desk II cold sputter/etch unit (Denton Vacuum LLC, Moorestown, NJ, USA). Images of SF/PCL composite nanofibrous mats were acquired using a Vltra55 (Carl Zeiss SMT Pte Ltd., Oberkochen, Germany) operating at 5 kV with a 16 mm working distance. The resulting images were imported into Image-Pro Plus software (ICube, Crofton, MD, USA) for analysis of nanofibrous diameter. One hundred fibers were analyzed.

The fiber orientation distribution (FOD) was obtained by the DHU-11 nanofiber orientation image analysis system (Shanghai Beiang Scientific Instruments Co., Ltd., Donghua University, Shanghai, China). This system can collect a series of images in different layers that cover the depth of the target with automatic focusing. Afterward, it can conduct fiber edge image processing and refining on the collected image information of the materials. Furthermore, it can automatically calculate the orientation of fiber webs within 1–180° based on a curvilinear integral algorithm. Finally, the original data of orientation distribution and the distribution curve can be obtained. Related research has shown that image fusion technology can be used to accurately measure the FOD of fiber mats [40,41].

Porosity was determined by finding the ratio of the measured mass of the specimen with the mass of a fully dense specimen of the same size by measuring the thickness, width, and length of the specimen. It has been reported that this method provides results that are similar to a mercury porosimeter method, which has been detailed by Rutledge et al. [42,43].

### 2.3. Mechanical Characterization

**Figure 2:**
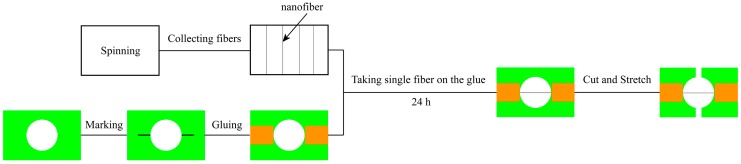
The flow chart for the preparation of single fiber tensile sample.

Uniaxial tensile tests were conducted on both electrospun single fibers and on the mats as a whole. SF/PCL nanofibers were deposited across a rectangular cardboard frame. A flow chart for the preparation of single fiber tensile sample is shown in Figure 2. First, a 6 mm hole is punched in a rectangular cardboard; the two ends of the hole are then marked, and glue is applied on the top; in the meantime, a single fiber with a metal frame is collected with the nanofiber collector for 1 s; in the end, the single fiber on the metal frame is placed on the glue at both ends of the cardboard and kept in a standard environment for 24 h to fix the single fiber on the cardboard. The frame containing a single nanofiber was mounted on the nano-mechanical stretching system (Agilent UTM T150, Santa Clara, CA, USA). Tensile testing of the single fiber was conducted at a constant engineering strain rate of 0.001 s^−1^. The initial gauge length used was 6 mm. Results are presented for twenty samples.

The tensile strength and the material strain against the tensile strength of mats were determined according to the ISO 527-1 [44] and ISO 527-3 [45] standard test methods. Electrospun nanofibrous mats were mounted on the uniaxial tensile stage with a gauge length of 30 mm in a KES-G1 multifunctional tensile test apparatus (Kato-Tech Company, Kyoto, Japan). The mats were cut into rectangular specimens that were 40 mm long and 30 mm wide using a razor guided by a straight edge. The thickness of each specimen was determined from the average of five measurements using a film thickness gauge (Shanghai Sixling instrument Factory, Shanghai, China). The specimens were stretched at a constant engineering strain rate of 0.01 s^−1^. Results are presented for five samples.

### 2.4. Tensile Analysis of Single Fibers

**Figure 3:**
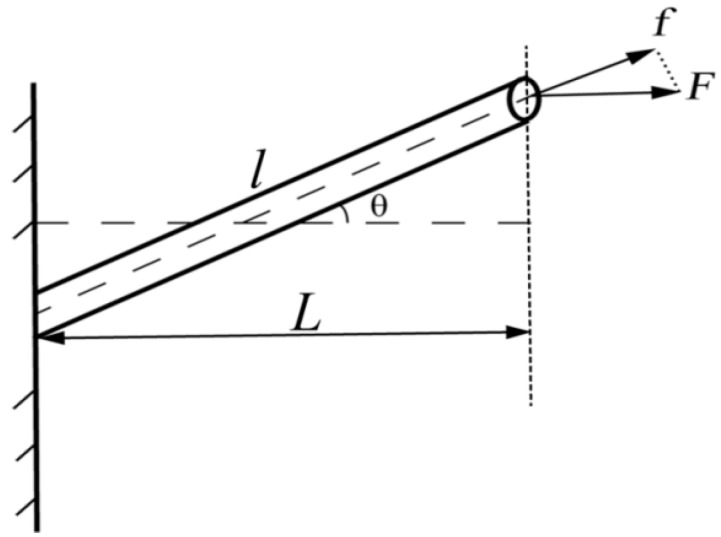
Single fiber stretched-schematic diagram.

As shown in Figure 3, the fiber is drawn with θ angle to the tensile force direction. The tensile force is

(1) $$f=\frac{F}{cos\theta }$$

where *F* is the applied tensile force, and *f* is the tensile force on the fiber.

Following this, the tensile force on the fiber and strain have a simple linear relation:

(2) $$f=\frac{E_{0}A}{l}\delta$$

where δ is a displacement in the direction of the fiber, l is the length of the fiber, $E_{0}$ is the elastic modulus of the single fiber, and A is the cross-sectional area of the fiber.

The relationship between the fiber displacement and the displacement of the tensile direction is as follows:

(3) $$\delta =\sqrt{{\left(lsin\theta \right)}^{2}+{\left(\delta_{0}+lcos\theta \right)}^{2}}-l$$

where $\delta_{0}$ is the displacement in the stretch direction.

When it is close to zero, the following can be solved:

(4) $$\frac{d\delta }{d\delta_{0}}=cos\theta$$

where *l* is fiber length, and its relationship with *L* is

(5) $$l=\frac{L}{cos\theta }.$$

Equations (4) and (5) are substituted into Equation (2), before the result is substituted into Equation (1), to obtain the following:

(6) $$F=\frac{E_{0}A}{L}cos^{3}\theta \;\delta_{0}.$$

## 3. Experimental Results

### 3.1. Single Fibers

**Figure 4:**
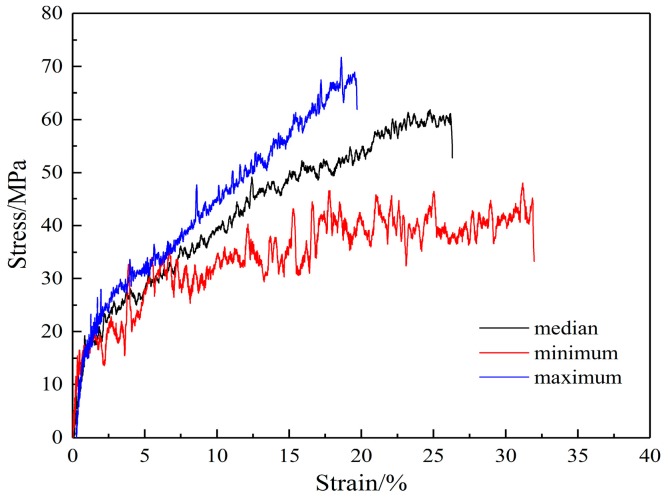
Stress-strain curves of a single fiber.

Single fiber stress-strain curves are shown in Figure 4. Only a small variation was found in the elastic modulus among fibers, although the yield stress, break stress, and break strain differed from fiber to fiber. The mechanical properties of the SF/PCL single fiber are given in Table 1. The variation in mechanical properties arises from variations in the molecular chain alignment, which occurs during the electrospinning process. The fibers that consist of better aligned chains exhibit greater strain hardening, which will allow them to reach maximum chain extension and fracture at lower strains than those that are less well aligned.

### 3.2. Mats Characterization

**Figure 5:**
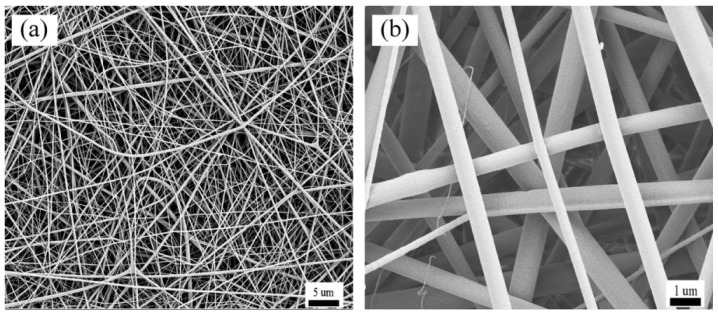
FE-SEM images of electrospun silk fibroin (SF)/polycaprolactone (PCL) nanofibrous mats. (**a**) 2000× magnification (scale bar = 5 microns); (**b**) 10,000× magnification (scale bar = 1 micron).

**Figure 6:**
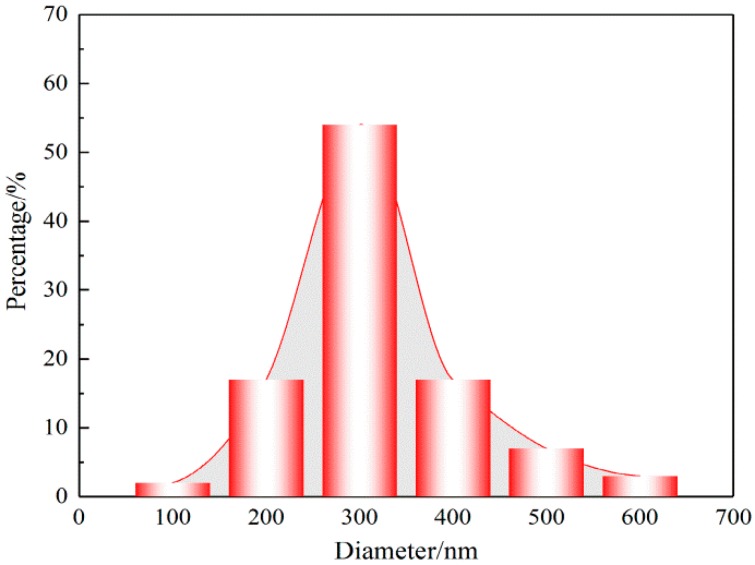
Diameter distribution of SF/PCL nanofibers.

**Figure 7:**
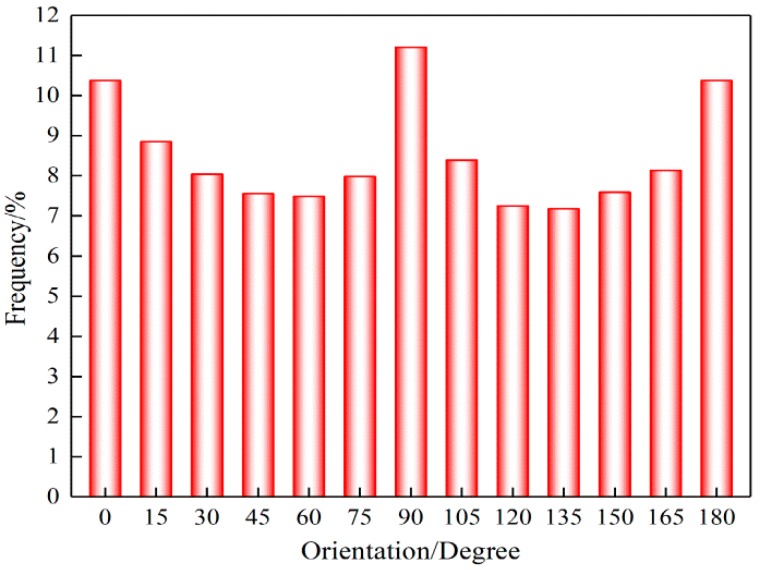
Fiber orientation distribution (FOD) in SF/PCL nanofibrous mats.

Using the methods described in the materials (Section 2.1), the FE-SEM images of the prepared electrospun SF/PCL nanofibrous mats are shown in Figure 5. The nanofibers were rod-shaped. The surfaces of the fibers were smooth without beads. Furthermore, there was no bonding at the intersection of fibers. Aided by the Image-Pro Plus images, the diameter of fibers was found to be mainly 300 nm (Figure 6). The diameter of fibers had an average of 272 nm and a standard deviation of 80 nm. To understand the distribution of nanofibers in mats, FOD was analyzed using the DHU-11 nanofiber orientation image analysis system. The results showed that the FOD approximated a uniform distribution (Figure 7).

**Figure 8:**
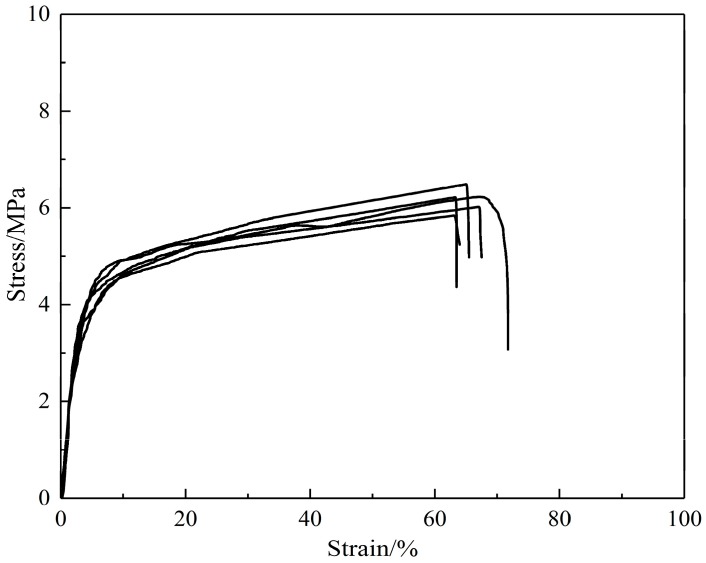
Stress-strain curves of SF/PCL nanofibrous mats.

The SF/PCL nanofibrous mats were prepared to have a porosity of 0.75. The specimens had a square shape. They were stretched at a constant engineering strain rate of 0.01 s^−1^. Five specimens were tested, and the mechanical properties are shown in Table 2. The tensile curves are shown in Figure 8.

## 4. Tensile Constitutive Relationship

The mechanical properties of the electrospun SF/PCL nanofibrous mats are determined by both the properties of single fibers and the microstructure of the mats. A continuous model was developed to reflect the microstructure of the nanofibrous mats. Fiber behavior is modeled as elastic-plastic based on single fiber experimental data.

In order to simplify the modeling process and improve calculation efficiency, a bi-linear assumption is needed to predict the mechanical properties of single fibers and whole SF/PCL nanofibrous mats [46].

### 4.1. Straight Fibers

After the relationship between the single fiber tensile force and the included angle of displacement relative to tensile direction was obtained, the average was determined through fiber integrals in various directions to obtain the relationship between the whole fiber web force and displacement, as the fiber web is uniform in various directional angles.

For a single fiber, we can obtain

(7) $$A=cos\theta A_{0}$$

where $A_{0}$ is an equivalent cross-sectional area of an oblique fiber in the tensile direction of the fiber web. The following can be solved by substituting it into Equation (6):

(8) $$F=\frac{E_{0}A_{0}}{L}cos^{4}\theta \delta_{0}$$

(9) $$\sigma_{0}^{\prime }=\frac{F}{A_{0}}\;\sigma_{0}^{\prime }=E_{0}cos^{4}\theta \varepsilon_{0}^{\prime }$$

where $\sigma_{0}^{\prime }$ is an equivalent stress of an oblique single fiber, and $\varepsilon_{0}^{\prime }$ is an equivalent strain of a single fiber. Therefore, we can obtain

(10) $$E_{0}^{\prime }=E_{0}cos^{4}\theta$$

where $E_{0}^{\prime }$ is an equivalent elastic modulus of a single fiber.

It is assumed that the fiber has a uniform distribution in all directions. Using this assumption, the elastic modulus of mats can be calculated according to the following integral:

(11) $$E_{m}^{e}=E_{0}\left(1-P\right)\frac{2\int_{0}^{\frac{\pi }{2}}cos^{4}\theta d\;\theta }{2\int_{0}^{\frac{\pi }{2}}d\;\theta }\;E_{m}^{e}=\frac{3}{8}E_{0}\left(1-P\right)$$

where 1 − *P* is the volume fraction of the fibers.

### 4.2. Curved Fibers

Up to this point, all fibers have been assumed to be straight, responding to an imposed deformation by changing length and rotating in response to the applied force. However, electrospun nanofibers were observed by scanning electron microscopy (SEM) to have some degree of curvature [27,47]. Such fibers can respond to deformation by bending or unbending. A modified version of the foregoing model includes consideration of bending or unbending on the change of fiber stiffness [48].

With reference to previous studies [48,49], the elasticity modulus of bending fiber web can be derived as follows:

(12) $$E_{m}^{e}=\frac{3}{8}SR\times E_{0}\left(1-P\right)$$

The bending correction coefficient is added in the elasticity modulus of the straight fiber web where *SR* is the stiffness ratio of the bending fiber and the straight fiber.

### 4.3. Elastic-Plastic Constitutive Model of Fibrous Mats

The modulus of single fiber can be expressed as follows:

(13) $$E_{f}=\{\begin{array}{l} E_{0},\;\sigma_{0}\leq \sigma_{0}^{y}\\ pE_{0},\;\sigma_{0}>\sigma_{0}^{y} \end{array}.$$

The modulus of fibrous mats can be expressed as such:

(14) $$E_{m}=\{\begin{array}{l} E_{m}^{e},\;\varepsilon_{m}\leq \varepsilon_{m}^{y}\\ E_{m}^{\prime },\;\varepsilon_{m}>\varepsilon_{m}^{y} \end{array}$$

According to the force analysis of the fiber mats, *θ* is very important. As the tensile stress of the mats increases, zero angular fiber will firstly enter the plastic phase, followed by $\theta_{1}$ angular fibers and finally $\frac{\pi }{2}$ angular fibers. When the $\theta_{1}$ angular fiber enters the plastic phase, the mats’ stiffness consists of two parts. Fibers that are greater than $\theta_{1}$ are still in the elastic phase, while the others that are smaller than $\theta_{1}$ are in the plastic phase.

(15) $$\frac{E_{m}^{\prime }}{E_{m}^{e}}=\frac{\int_{\theta_{1}}^{\frac{\pi }{2}}cos^{4}\theta d\;\theta +p\int_{0}^{\theta_{1}}cos^{4}\theta d\;\theta }{\int_{0}^{\frac{\pi }{2}}cos^{4}\theta d\;\theta }.$$

The tensile stress of a single fiber is

(16) $$\sigma_{0}=E_{0}cos^{2}\theta \;\varepsilon_{m}.$$

For the $\theta_{1}$ angular fiber, the strain of the fibers in the mats can be written as

(17) $$cos^{2}\theta_{1}=\frac{\varepsilon_{m}^{y}}{\varepsilon_{m}}.$$

The stress-strain curve can be obtained by integrating the modulus of the plastic segment.

## 5. Results and Discussion

### 5.1. Finite Element Modeling

Relative to atomic spacing (10^−1^ nm), the fiber diameter is larger. It can meet the continuous medium hypothesis and can be simulated through the finite element method.

Msc.Nastran 10.0 software from MSC (Newport Beach, CA, USA) was used for finite element computation, while the modeling and results analysis were carried out through Hypermesh/Hyperview 12.0 software from Altair (Troy, MI, USA). For the convenience of modeling, μm was used as the length unit, μN as the force unit, and MPa as the stress unit in Msc.Nastran. Fiber diameter was set at 0.272 μm, and length was 6 μm. It was modeled into Msc.Nastran bean elements, with a single fiber divided into 60 elements and an element length of 0.1 μm.

It was assumed that the material was isotropic. The elasticity modulus $E_{0}$ was set at 1717 MPa. Poisson’s ratio υ was set at 0.3. The fiber was a solid cylindrical structure. The porosity of the SF/PCL nanofibrous mats was measured to be 74.12–77.75%. It was taken as 75% going forward. According to the geometric structure of nanofibrous mats, a finite element model was established. It was assumed that the quantity of fibers was 1000 to make the overall element size within the 100,000 scale. The number of elements in each fiber needed to be smaller than 100 with 100 beam elements, so simulation size was set as 100 μm × 100 μm.

(18) $$1-P=N\times \pi \times {\left(D/2\right)}^{2}/10000/T$$

where P is the porosity of the mats, *N* is the number of units, *D* is the fiber diameter, and *T* is the thickness of the mats.

**Figure 9:**
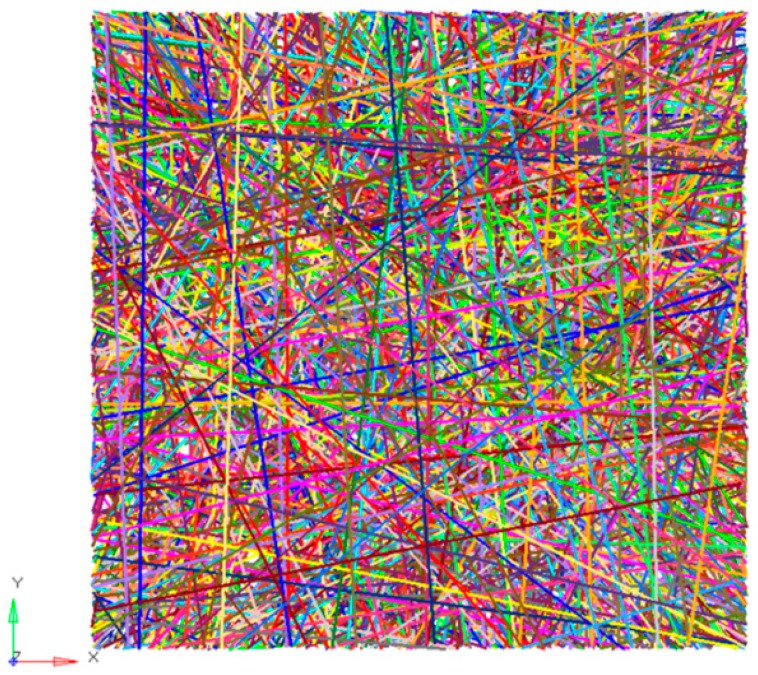
Finite element analysis model of nanofibrous mats.

In the 100 × 100 μm grids, 1000 straight fibers were distributed randomly, which was generated through Matlab R2012a programming (Natick, MA, USA). The analysis model of the nanofibrous mats is shown in Figure 9. There were 93,871 actually generated elements, and its thickness was 2.27 μm.

### 5.2. Parameter Analysis

For the experimental loading in the simulation, 123 degrees of freedom were constrained for all nodes in respective 5 μm regions at left and right ends with a tensile elongation of 0.9 μm. Meanwhile, as some fibers were in up and down directions, to clamp left and right sides, only one side could be clamped or neither could be clamped. These types of fibers would result in a singularity of the stiffness matrix, so these fibers were deleted (these fibers would not influence the tensile modulus in left and right directions).

#### 5.2.1. Length-Width Ratio Parameter

**Figure 10:**
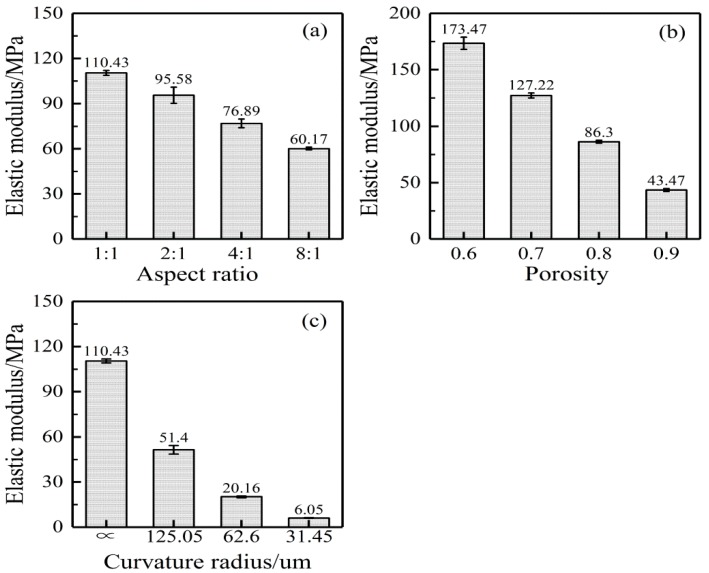
Effect of the structural parameters on the elastic modulus of nanofibrous mats. (**a**) length-width ratio; (**b**) porosity; (**c**) fiber curvature.

The length-width ratio influences the tensile results of the nanofibrous mats. Three groups of models were established for the tensile simulation test, with each group having four situations. The length-width ratios of mats were respectively 1:1, 2:1, 4:1, and 8:1. All models had the same porosity (75%), and the results are shown in Figure 10a. With an increase in the length-width ratio, the elasticity modulus decreased. When it was 8:1, the elasticity modulus was about half of that under 1:1.

#### 5.2.2. Porosity

Porosity will also affect the tensile results of the nanofiber mats. Three groups of models were established for a tensile simulation test, with each group having four situations. The porosities of the nanofibrous mats were respectively 60%, 70%, 80%, and 90%. All models had the same length-width ratio (1:1), and the results are shown in Figure 10b. Porosity had a significant influence on the tensile elasticity modulus of the nanofibrous mats.

#### 5.2.3. Fiber Curvature

**Figure 11:**
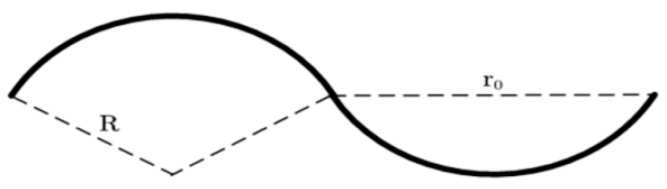
Schematic diagram of a bend fiber. Here, *R* and $r_{0}$ are the radius and wavelength of the sinusoidal curve.

It was originally assumed that the fibers in the mats were straight. However, they actually had certain curvatures (Figure 5a). Therefore, a certain curvature was introduced based on straight fibers for the tensile test. It was assumed that fibers had a thickness in the *Z*-direction, as shown in Figure 11. When the $r_{0}$ was fixed at 10 μm, there were 5 cycles in 100 μm. When 0.1, 0.2, and 0.4 were taken as fiber heights, the radiuses of the curvatures were 125.05, 62.60 and 31.45.

**Figure 12:**
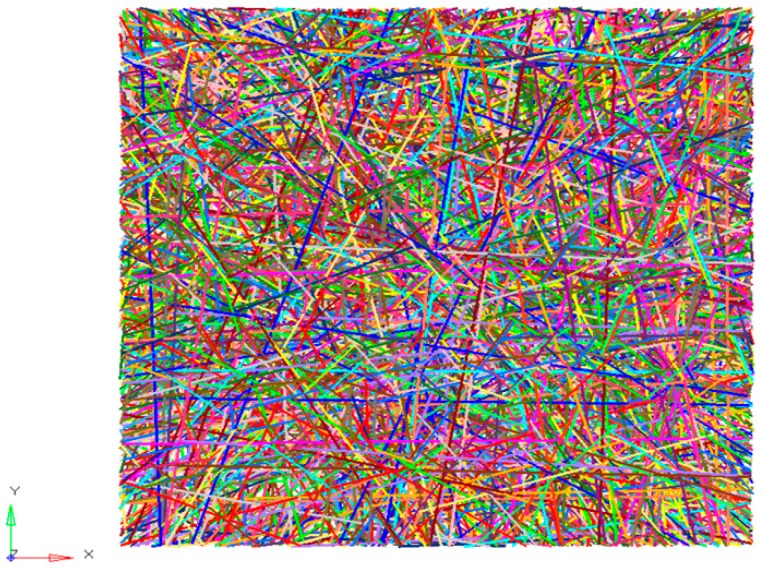
Finite element model of bend fibers.

In the 100 × 100 × 2.27 μm grids, a thousand bend fibers distributed randomly on the *X*-*Y* plane with a curvilinear distribution in the *Z*-direction. The established finite element model of bend fibers is shown in Figure 12. Three groups of models were simulated and a group had four fiber curvatures. All models had the same length-width ratio (1:1) and porosity (75%). All results are shown in Figure 10c. It shows that, with a decrease in the radius of the curvature, elasticity modulus had an obvious decreasing trend. During the tensile process, bend fibers would be straight without elongation. Therefore, they had little contribution to the tensile mechanical properties of the nanofibrous mats.

### 5.3. Model Validation

**Figure 13:**
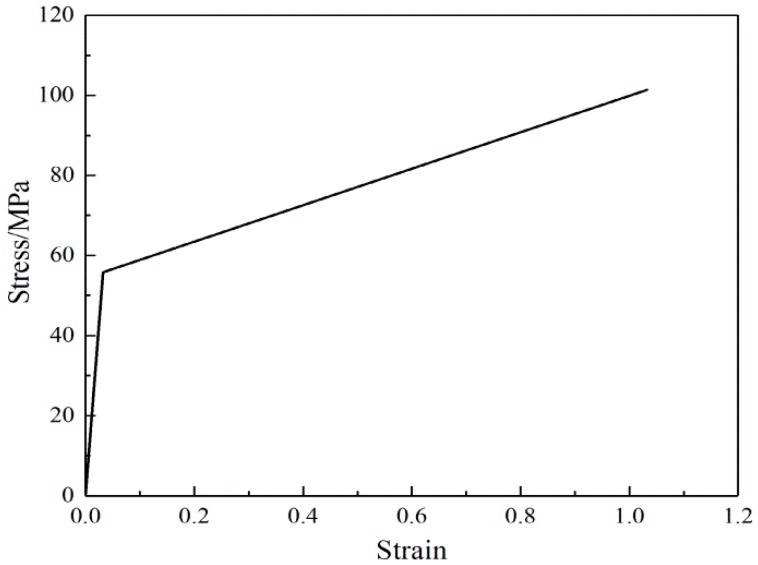
Stress-strain curve of single fiber.

To further assess the predictive capability of the constitutive model, uniaxial tensile loading experiments were conducted under additional conditions (aspect ratios = 1; porosity = 75%). The model was calculated and analyzed using Abaqus 11.3 software (Providence, RI, USA). Based on theoretical analysis of the fiber web and fiber linear elasticity, fiber deformation was directly related to fiber web deformation, and elasticity modulus of the fiber web was in direct proportion to that of the single fibers, as shown in Equation (12). Therefore, if the stress-strain curve of fiber web is bi-linear, the fiber material parameters should also be bi-linear. It was assumed that the fibers presented a linear stress-strain response after yielding, while the stress-strain ratio to that in the elastic phase was the same as the ratio in the nanofibrous mats (2.6:98). The stress-strain curve of a single fiber is shown in Figure 13.

**Figure 14:**
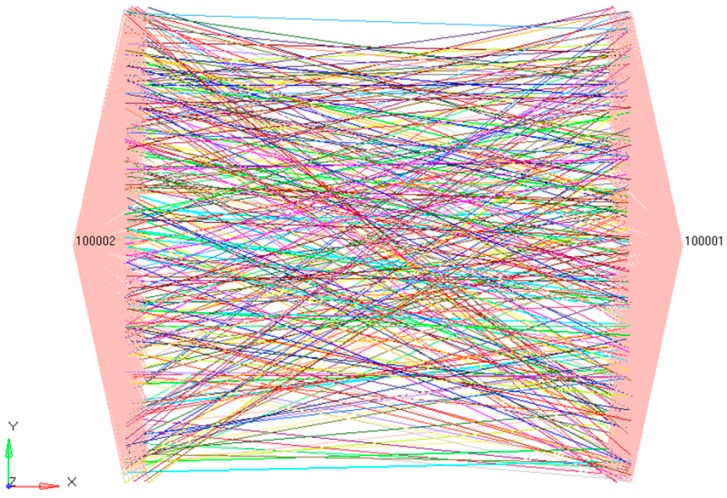
Finite element loading diagram of nanofibrous mats.

**Figure 15:**
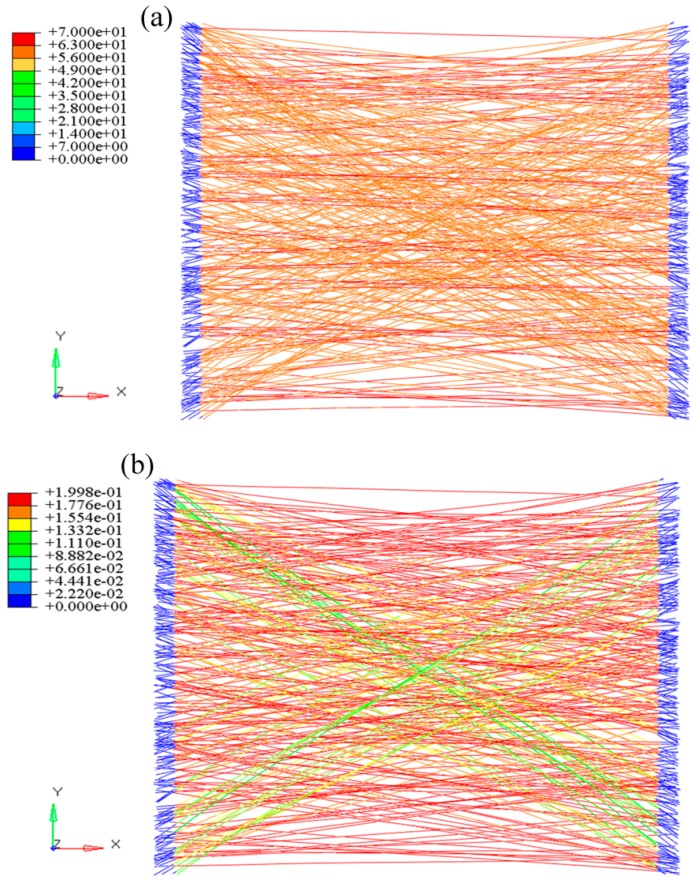
Tensile cloud image of nanofibrous mats. (**a**) stress (MPa); (**b**) strain.

For the convenience of extracting the stress and displacement of the loading process, loading nodes at the left and right were connected to Nodes 100001 and 100002 (Figure 14). After this, 18 μm of displacement was applied to Node 100001 (the corresponding strain of the fiber web was 0.2). The analysis was set as 20 steps and solved through the static/general method. The calculation results of the elastic-plastic nanofibrous mats are shown in Figure 15. It shows that, after entering the plastic phase, the fiber web modulus decreased to be 2.7% of that in the linear segment. The stress distributions of the fiber web were more uniform.

Since we already know the single fiber’s elastic modulus $E_{0}$ and the fiber web’s fiber volume fraction 1 − P, then according to the derived Equation (11), we can calculate the fiber web’s initial elastic modulus $E_{m}^{e}$. When the mats go into the stage of plastic tension, as shown in Equation (14), the total stress of the mats should be elastic section stress add the plastic section stress, and the plastic section stress is calculated by the following steps: 1. $\theta_{1}$ is determined by the fiber web’s yield stress and strain and applied to Equation (17). 2. Since the indefinite integral $\int cos^{4}\theta d\theta =\frac{3}{8}\theta +\frac{1}{4}sin2\theta +\frac{1}{32}sin4\theta +C$, the value of $\theta_{1}$ is applied to Equation (15), where the proportional coefficient *p* is determined by the strengthening modulus and initial elastic modulus of the mats, $p=\frac{E_{m}^{\prime }}{E_{m}^{e}}$ and the section area under the plastic stress-strain curve can be calculated following the change of $\theta_{1}$. 3. The segmented area is summed to obtain the plastic stress. The elastic stress can be seen from the simple stress-strain relationship $\sigma =E_{m}^{e}\varepsilon$, so the theoretical tensile stress-strain curve of the fiber web can be obtained.

**Figure 16:**
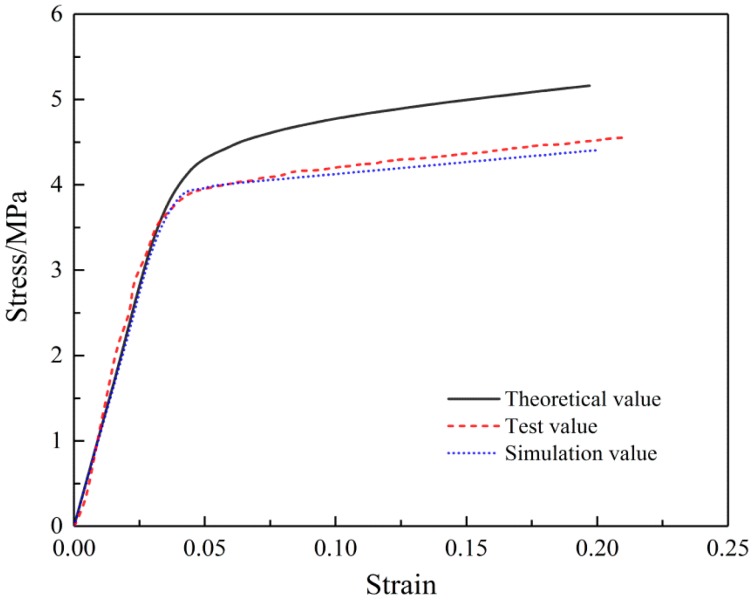
Comparison of the tensile stress-strain curves.

Experimental stress-strain curves of SF/PCL nanofibrous mats compared with theoretical analysis and simulation results are shown in Figure 16. The simulation results are consistent with the experimental results, but the theoretical value was greater. Through analysis, we found that theoretical analysis and calculation contained fiber tensile contributions in all directions, while experimental measurement specimens and simulation models truncated some fibers.

## 6. Conclusions

The nanofibers were introduced directly into the model according to their FOD. This direct microstructure-based numerical approach maintains the relation of the microstructure of electrospun nanofibrous mats with its deformation and damage behavior. Moreover, this technique introduced porosity in the microstructure of the mats, which cannot be achieved with a traditional continuous model.

All parameters needed in simulation were obtained through single fiber and fibrous mat experiments, including orientation distribution function, geometric properties, and material properties. The developed model reproduced the deformation of fibrous mats and gradual damage mechanisms. Meanwhile, the theoretical tensile constitutive relation reflected the geometric properties of the single fibers (straight and bending) and geometric properties (shape and porosity) of the nanofibrous mats. Compared with the experiments, the accuracy of the tensile constitutive relation and the developed model was verified. The constitutive relation and the model were found to be in good agreement with the experiments in terms of the deformed shape of specimens and the stress-strain curves and reproduced all of the main features of mat deformation, including

- a greater length-width ratio and porosity of specimen results in a lower elasticity modulus of nanofibrous mats; a greater number of bending fibers in nanofibrous mats is related to a smaller radius of curvature and a lower elasticity modulus of nanofibrous mats;
- elastic-plastic axial stress-strain behavior, including elasticity modulus and yield modulus;
- a re-orientation of nanofibers toward the loading direction, which is anisotropic behavior;
- a smaller included angle between the single nanofiber and tensile loading direction, which occurs before the fiber entered the plastic deformation zone.

The developed model using the parametric modeling technique provides an opportunity for a new study of the effects of variation in the nanofibrous mat’s geometry on its overall deformation. Furthermore, the effect of the geometry of fibers and mats can be studied using this model. This model can capture the anisotropic stress-strain behavior of the material and provide an insight into specific features of deformation of fibers and the mat. The progressive damage mechanism can also be observed during the simulation process. Such capabilities of the model would help to understand the behavior of electrospun nanofibrous mats and its structure-property relationships. This constitutive relation and model aids in the design of electrospun nanofibrous mats used in engineering applications.

## Figures and Tables

**Table 1:** Mechanical properties of the silk fibroin (SF)/polycaprolactone (PCL) single fiber.

Property	Average	Minimum	Maximum	CV
$E_{0}$ (MPa)	1717	1550	1900	0.09
$\sigma_{0}^{y}$ (MPa)	18	15	23	0.10
$\sigma_{0}^{b}$ (MPa)	60	43	68	0.17
$\varepsilon_{0}^{b}$	0.26	0.20	0.32	0.17

**Table 2:** Mechanical properties of SF/PCL nanofibrous mats.

Property	Average	Minimum	Maximum	CV
$E_{m}^{e}$ (MPa)	98	75	120	0.16
$\sigma_{m}^{y}$ (MPa)	3.9	3.2	4.4	0.12
$\varepsilon_{m}^{y}$	0.049	0.044	0.052	0.06
$E_{m}^{\prime }$ (MPa)	2.6	2.3	3.0	0.11
$\sigma_{m}^{b}$ (MPa)	6.14	5.83	6.49	0.04
$\varepsilon_{m}^{b}$	0.665	0.635	0.715	0.06

## Nomenclature

$\sigma_{0}^{y}$	yield stress of single fiber (MPa)	$A_{0}$	equivalent cross-sectional area of single fiber (μm^2^)
$\sigma_{0}^{b}$	break stress of single fiber (MPa)	$\sigma_{0}^{\prime }$	equivalent stress of single fiber (MPa)
$\varepsilon_{0}^{b}$	break strain of single fiber (%)	$\varepsilon_{0}^{\prime }$	equivalent strain of single fiber
$E_{m}^{e}$	initial elastic modulus of mats (MPa)	$E_{0}^{\prime }$	equivalent elastic modulus of single fiber (MPa)
$\sigma_{m}^{y}$	yield stress of mats (MPa)	*P*	porosity of mats (%)
$\varepsilon_{m}^{y}$	yield strain of mats (%)	1 − *P*	volume fraction of fibers (%)
$E_{m}^{\prime }$	strengthening modulus of mats (MPa)	*SR*	stiffness ratio
$\sigma_{m}^{b}$	break stress of mats (MPa)	$E_{f}$	modulus of single fiber (MPa)
$\varepsilon_{m}^{b}$	break strain of mats (%)	$\sigma_{0}$	stress of single fiber (MPa)
F	tensile force (μN)	$E_{m}$	modulus of mats (MPa)
f	tensile force on single fiber (μN)	$\varepsilon_{m}$	strain of mats (%)
θ	angle between the fiber and the stretching direction (°)	*N*	number of units
δ	displacement in the direction of the fiber (μm)	*D*	fiber diameter (μm)
l	length of single fiber (μm)	*T*	thickness of mats (μm)
*A*	single fiber cross-sectional area (μm^2^)	*R*	curvature radius (μm)
$E_{0}$	elastic modulus of single fiber (MPa)	*k*	aspect ratio of mats
$\delta_{0}$	displacement in the stretch direction (μm)	υ	Poisson’s ratio
*L*	length (μm)	*p*	Ratio coefficient
